# The Assessment Methods of Laryngeal Muscle Activity in Muscle Tension Dysphonia: A Review

**DOI:** 10.1155/2013/507397

**Published:** 2013-11-04

**Authors:** Seyyedeh Maryam Khoddami, Noureddin Nakhostin Ansari, Farzad Izadi, Saeed Talebian Moghadam

**Affiliations:** ^1^Department of Speech Therapy, School of Rehabilitation, Tehran University of Medical Sciences, Enghelab Avenue, Pitch-e-shemiran, Tehran 11489, Iran; ^2^Department of Physiotherapy, School of Rehabilitation, Tehran University of Medical Sciences, Tehran, Iran; ^3^Department of Ear, Nose, Throat, Head and Neck Research Center, Hazrat-e-Rasoul Hospital, Tehran, Iran

## Abstract

The purpose of this paper is to review the methods used for the assessment of muscular tension dysphonia (MTD). The MTD is a functional voice disorder associated with abnormal laryngeal muscle activity. Various assessment methods are available in the literature to evaluate the laryngeal hyperfunction. The case history, laryngoscopy, and palpation are clinical methods for the assessment of patients with MTD. Radiography and surface electromyography (EMG) are objective methods to provide physiological information about MTD. Recent studies show that surface EMG can be an effective tool for assessing muscular tension in MTD.

## 1. Introduction

Muscular tension dysphonia (MTD) is a common functional dysphonia manifested by excessive tension in the intrinsic and/or extrinsic (para) laryngeal muscles. Approximately 10–40% of the clients at a voice clinic have MTD [1–3]. The etiology of MTD is multifactorial, which leads to voice disturbance. Women in middle age are predominantly affected. The musculoskeletal tension is a core feature of the MTD [4–8].

The pathophysiology of MTD is not fully understood [9]. In the presence of MTD, the tension of extrinsic muscles is altered, which moves the larynx high in the neck and disturbs the inclination of the cartilages of the larynx. Consequently, the intrinsic muscles of the larynx are affected. In this way, vocal folds tension is changed and causes the voice disturbance [10].

The assessment of extrinsic laryngeal muscular tension is important for the diagnosis of voice disorders [11]. There are different assessment methods available to document treatment outcome and to record the laryngeal muscle tension in MTD [12]. There are subjective and objective measures to examine patients with MTD. The commonly used methods are clinically based techniques such as history taking, palpation, and musculoskeletal assessments. However, such clinical measures are prone to subjectiveness. Recently, surface EMG has been used as an objective instrument to measure the tension of laryngeal muscles in patients with MTD. This paper aimed to review the literature on common assessment methods of measuring muscle tension in MTD to present their advantages and disadvantages and identify the best tools for practice and research.

## 2. Examination of MTD

Assessment methods of MTD can be classified into two distinct groups: (1) noninstrumental methods, which do not need any equipment for examination (e.g., case history, palpation); (2) instrumental methods, which use tools for objective diagnosis of conditions and include observation, radiography, and electromyography.

## 3. Noninstrumental Methods

### 3.1. Case History

Case history is a routine and simple clinical method to assess muscular tension in patients with MTD. Patients are usually asked for vocal misuse/abuse and influences of the stress or psychological factors on voice [7, 13]. Pain in larynx or around the larynx is an important symptom associated with MTD [14]. Though simple, case history is subjective; this needs confirmation by palpation or objective methods.

### 3.2. Palpation

Palpation of neck is a routine procedure for the evaluation of muscular tension around the larynx [10]. Elevation of larynx is one of the core features of MTD, which can be effectively evaluated by palpation [5, 7, 12, 15–17]. However, palpation is a subjective method for which only a few standardized scales have been developed [10, 11, 18, 19]. Angsuwarangsee and Morrison established a clinical evaluation technique for examination of extrinsic laryngeal muscular tension by palpation. They assessed laryngeal tension using a 4-point scale at rest as well as phonation and found it useful for diagnosis of muscle tension dysphonia and internal laryngeal postures [10]. Kooijman et al. used a similar technique; the target muscles tested were different (extralaryngeal elevators, laryngeal tensor, and head and neck muscles; laryngopharyngeal muscles were not included) and the judgments performed only at rest [11]. Mathieson et al. used neck palpation to assess laryngeal manual therapy outcomes and determined the degree of muscle resistance using a 5-point scale and the height of the larynx in the vocal tract at rest. They showed changes in the laryngeal position which was not confirmed by acoustic data [18]. A recent study to examine the interrater reliability and validity (correlation with sEMG) of palpation rating systems of Angsuwarangsee and Morrison [10] and Mathieson et al. [18] when administered by speech-language pathologists unfamiliar with these scales found low reliability and validity. The authors concluded that these scales though helpful for voice therapists, who are beginners, may not be sensitive tools to assess changes occurred following individual treatment [19].

*Advantages and Disadvantages.* Palpation is a clinical, easy used method to assess muscular tension. This technique needs no special equipment for use in clinical practice. However, it is a subjective method because it is based on ratings reducing the measurement reliability. Another problem with palpation is the lack of standard criteria for its use. Furthermore, there are no sufficient data about the psychometric properties (e.g., reliability, validity, and sensitivity) of available rating systems [10, 18–20]. Reliability studies of rating systems for assessing muscle tension show that a poor interrater reliability can be obtained. For rating systems available, there are missing data on the test-retest reliability. Nevertheless, the therapists prefer to use qualitative neck palpation protocols in clinical practice.

## 4. Instrumental Assessments

### 4.1. Observation

Observation of larynx and vocal folds is a critical part of voice examination. There are some criteria that can be used as the primary diagnostic indicators in MTD. The key features of MTD include posterior glottal chink, mucosal vocal folds changes, suprahyoid muscle tension, hard glottal attack, and larynx rise [13]. The investigations to evaluate the diagnostic values of the above criteria have demonstrated that not all of them can exactly distinguish the patients with MTD from normal ones [21–27]. The hyoid and laryngeal positions have been shown to be higher in patients with primary MTD compared to subjects without voice disorders [20]. It has been suggested that the larynx elevation may increase the anterior-posterior (A-P) supraglottic contraction [11] which in turn can lead to improper vibratory patterns [28]. Information about normal vocal fold mucosa is essential in distinguishing functional voice disorders such as MTD from subtle vocal fold lesions [10]. There are no specific mucosal changes in primary MTD. However, the vocal fold nodules, polyps, and cysts found in MTD have been most common mucosal changes observed in this pathology [12]. Nevertheless, patients may use compensatory hyperkinetic laryngeal behaviors to achieve glottal closure. In this way, any underlying organic condition such as presbylaryngis or vocal folds paresis may be overlooked. This type of MTD can be referred to as secondary MTD resulting from a patient's compensation to an underlying organic disease [21]. A study by Paoletti et al. found heterogeneity in the laryngeal features in telemarketers with MTD and their presence among control subjects suggesting that they cannot help to diagnose the MTD [22]. Investigators have reported that subjects with hyperfunctional voice may have static components of false vocal fold and anterior-posterior (A-P) contractions [23]. Furthermore, the supraglottic activity has a role in normal speech production and should not necessarily be considered the excessive muscle tension [24]. It should be noted that although the A-P compression has been observed in greater degree in dysphonics it is also a common finding in normal subjects. In addition, the medial compression of the ventricular folds has been reported to be as a normal laryngeal posture [25].

A recent investigation by stepp et al. questioned the use of some measures such as the estimates of AP supraglottal compression, quantitative measures of AP, and false vocal fold (FVF) supraglottal compression [26]. In order to determine whether the frequency of hard glottal attack (HGA) was different in hyperfunctional voice patients with and without vocal fold masses, Andrade et al. found that all groups with voice disorder demonstrated higher frequencies of HGA than the control group, and there were differences between the male and female subjects. They reported no differences between the various disorders [27].

*Advantages and Disadvantages.* Larynx observation is the most common practical technique used in assessing all voice disorders to show muscle tension, but this technique lacks sufficient discriminate validity to distinguish effectively the MTD from normal condition. Among others, the larynx rise and vocal nodule can be considered as the key diagnostic signs in MTD. Equipments may be used by the practitioners for the observation but it essentially is based on the examiner perception. Furthermore, observational method of rigid endoscopy may activate gag reflex and induce supraglottic constriction [14]. Utility of observational methods is questioned in the diagnosis of MTD.

### 4.2. Radiography

Radiography can be used for differential diagnosis of MTD. In a study to determine whether radiographic measures for patients with primary MTD were different from those of normal subjects, Lowell et al. (2012) studied 10 patients with primary MTD and 10 normal subjects radiographically while producing phonation. They reported higher positions of the hyoid and larynx during phonation in MTD patients compared with normal subjects. This study indicates that radiographic measures targeting hyoid and larynx can be used in delimitation of pathologic patterns in MTD during phonation [20].

*Advantages and Disadvantages.* Radiography has improved the differential diagnosis of MTD. It can provide objective evidence for hyolaryngeal elevation in MTD. However, radiography is not available for routine clinical use in voice clinics, and patients are exposed to radiation. Furthermore, radiography is not sensitive enough to distinguish patients with MTD as some patients may show an asymmetric reduction in the hyolaryngeal space, and this space might not reliably reflect the differences in laryngeal elevation when radiographic images conducted through lateral cephalograms [20]. Further investigations with large sample size are needed to determine the usefulness of radiography.

### 4.3. Electromyography

Electromyography (EMG) of the larynx is a standard test to evaluate the integrity of muscular and nervous system of larynx thorough recording action potentials generated in the muscle fibers. The EMG technique may use needle or surface electrode for recording muscle activity. The needle EMG is an invasive technique in which electrodes are inserted into the target muscles. This procedure can be used reliably in diagnosis of voice problems associated with neurological or neuromuscular conditions [29]. An evidence-based review of laryngeal EMG demonstrated that the laryngeal EMG (LEMG) is useful for injection of botulinum toxin into the thyroarytenoid muscle in adductor spasmodic dysphonia, but there are no sufficient evidence-based data to support or refute it for other laryngeal disorders [30].

The surface EMG (sEMG) is used to record muscle activation using surface electrodes. As reported in the literature, sEMG can be used as an objective measure for diagnosis or outcome assessment in MTD [31–36]. The sEMG in the form of EMG biofeedback has been used in the treatment of patients with MTD [37–40]. Redenbaugh and Reich (1989) studied neck EMG levels in normal and vocally hyperfunctional speakers and found significantly higher EMG levels for hyperfunctional speakers [31]. It was first attempt to show sEMG in vocal hyperfunction. They detected sEMG in 7 normal and 7 vocally hyperfunctional speakers and found significant differences between the two groups on all EMG measures except for the resisted-force maneuvers, the vowel EMG-to-rest EMG ratio, and the speech EMG-to-rest EMG ratio [31]. Thereafter, Hočevar-Boltežar et al. in 1998 included 11 patients with MTD and 5 normal speakers to determine the EMG characteristics of muscles in the perioral area and anterior neck before and during phonation using 9 pairs of surface electrodes. Their results showed a 6–8-fold increase of EMG activity and/or an alternation of the EMG activity level in the perioral and supralaryngeal muscles patients with MTD [32].

Several investigations have been performed to determine the vocal hyperfunction behaviors in vocal fold paralysis, MTD, and vocal fold nodules using sEMG [19, 26, 33]. In a study to determine the sensitivity of the anterior neck sEMG to changes in vocal hyperfunction associated with injection laryngoplasty, the results did not support the use of sEMG measures for assessing vocal hyperfunction [26]. In an attempt to characterize phonatory function in singers and nonsingers with vocal fold nodule using sEMG, the authors concluded that the nodule morphology did not differ between the two groups [33]. Furthermore, to compare neck palpation rating systems (PRS) with sEMG, Stepp et al. (2011) examined a single session voice therapy outcomes in vocal hyperfunction in 16 participants with neck muscle tension. They concluded that the PRS were not sensitive tools for monitoring changes that might occur in muscle tension following treatment [19]. Recently, a study conducted by Van Houtte et al. to examine the sEMG for the assessment of MTD concluded that the sEMG was not able to detect an increase in muscle tension in patients with MTD and questioned the use of sEMG as a diagnostic tool for distinguishing patients with and without MTD [34].

Many factors should be taken into consideration when using sEMG for measurement of laryngeal hyperfunctional behaviors. These factors can be classified into vocal tasks, participant' characteristics, and factors affecting EMG recording outcomes. Speech tasks of connected speech or reading can distinguish patients with MTD from subjects without MTD compared with tests at rest or phonation tasks. The tension during connected speech changes quickly and flexibly, and it is restricted in the presence of tension [11, 33]. Age, gender, type of MTD (primary or secondary), and severity of voice disorders should also be considered. The subjects should be matched with regard to the age, type of MTD, and anatomical as well as the physiological laryngeal differences between males and females when preparing protocol for sEMG measurements. Other important factors are type of electrode (unipolar, bipolar, and double-differential electrodes), number of electrodes, normalization method, electrode location, outcome measure, and data analysis method (Table 1). The sEMG is mainly performed at the suprahyoid muscle group, the thyrohyoid, the cricothyroid, and the sternocleidomastoid (Table 1). The laryngeal elevation during phonation, high vocal pitch, and postural problems in MTD have been the reasons for considering muscles for sEMG recordings [10, 11, 20].

One important factor which should be kept in mind when using sEMG to quantify neck muscle tension is the variability due to the electrode contact and neck musculature of the subjects. Normalization procedure against a reference contraction can be considered as a way to overcome this problem but it is a difficult task in the assessment of speech muscles. Problems with amplitude normalization have led the researchers to suggest intermuscular coherence as a method to obtain reliable data when assessing vocal hyperfunction [35]. Coherence is a linear dependency between the two variables at special frequencies and measures strength of coupling between the two [36]. The beta band indicates a frequency of 15–35 Hz which originates mainly from primary motor cortex [37]. The Beta band coherence represents transmission from primary motor cortex to spinal motoneurons, with cortical-muscle links [38]. It is typically associated with production of static motor tasks [39]. The coherence in the study of speech and voice has not been extensively investigated.

Recently, sEMG was measured from two electrodes on the anterior neck surface of 18 subjects with vocal nodules and 18 subjects with normal voice to explore the intermuscular coherence in the beta band as a possible indicator of vocal hyperfunction. Coherence was calculated from sEMG data while subjects produced both read and spontaneous speech. The speech type had no significant effect on average coherence, and the mean coherence in the beta band was significantly lower than that in control group. Authors concluded that the EMG beta coherence in neck strap muscle during speech production can be an indicator of vocal hyperfunction [35]. To better understand the neck intermuscular beta coherence (NIBcoh) in healthy individuals, Stepp et al. (2011) measured mean NIBcoh using sEMG at 2 anterior neck locations in 10 subjects and found that mean beta intermuscular coherence reduced in mimicking a hyperfunctional voice [40] (Table 1).

*Advantages and Disadvantages.* An advantage of sEMG is that it provides objective and robust data on the muscle activity. A measure such as sEMG could become a valuable tool for therapists to assess reliably muscle tension in patients with MTD. However, the EMG is not available, needs equipment which is expensive, and needs training to use and interpret data. There are several reports that did not find it useful and there is no benchmarked normal for comparison.

## 5. Conclusion

Various assessment methods (clinical, radiological, and electromyography) have been used to measure laryngeal muscular tension in patients with MTD. The commonly used methods for evaluation and diagnosis of MTD are clinical, which includes case history, observational techniques, and palpation. The radiography as well as the sEMG can be used as objective measures for differential diagnosis of MTD. The evaluation of muscle activity using sEMG provides a measure to quantitatively obtain neurophysiological data in assessing MTD. Surface EMG with intermuscular beta coherence at frequency range of 15–35 Hz could be used to assess vocal hyperfunction. The researchers could use sEMG as a means to investigate the underlying physiological mechanisms involved in MTD.

## Figures and Tables

**Table 1:** Summaries of studies using surface electromyography.

Authors	Design	Participants	Tasks	Type of electrodes	Electrode positioning	Outcome measure	Results
Redenbaugh and Reich 1989 [31]	Case-control	7 normal and 7 patients with vocal hyperfunction	At rest, phonation, and reading	Unipolar	Thyrohyoid membrane	RMS	(i) EMG levels in MTD significantly higher than normal (ii) Moderately high correlations between clinical measures and speech EMG values

Hočevar-Boltežar et al. 1998 [32]	Case-control	5 normal and 11 patients with MTD	At rest, phonation	Unipolar	Perioral area and anterior neck	RMS	(i) Increases of EMG activity in the perioral and supralaryngeal muscles before and during phonation (ii) Same sEMG level for both groups at rest

Stepp et al. 2010 [26]	Pretest-posttest	13 patients with vocal folds paralysis (before and after thyroplasty injection)	Phonation, reading, and spontaneous speech	Double-differential	(1) Thyrohyoid, omohyoid, and sternohyoid (2) Cricothyroid and sternohyoid (3) SCM	RMS	(i) No significant reductions in RMS after injection (ii) No significant effects of after vocal tasks (iii) The largest changes associated with the electrode position 1

Stepp et al. 2011 [33]	Case-control	10 normal and 18 patients with vocal nodules (10 singers and 8 nonsingers)	Phonation, reading, and spontaneous speech	Double-differential	(1) Thyrohyoid, omohyoid, and sternohyoid (2) Cricothyroid and sternohyoid (3) SCM	RMS	(i) No significant difference between groups (ii) Significant effect of vocal tasks (iii) Useful for assessing inappropriate phonatory behaviors in nodules

Stepp et al. 2011 [19]	Pretest-posttest	16 patients with vocal hyperfunction (before and after one session voice therapy)	Phonation, reading, and spontaneous speech	Double-differential	(1) Thyrohyoid, omohyoid, and sternohyoid (2) Cricothyroid and sternohyoid (3) SCM	RMS	(i) No reliably changes over one session voice therapy (ii) Stronger relationship in suprahyoids in a smaller set of patients with vocal nodules

Van Houtte et al. 2013 [34]	Case-control	44 normal and 18 patients with MTD	At rest, phonation, and reading	Bipolar	(1) Mylohyoid, geniohyoid, and digastric (2) Sternohyoid and omohyoid (3) SCM	RMS	(i) Not able to discriminate between MTD and normal subjects (ii) Type of electrodes, nature of primary MTD, and emotional state of the subjects as important factors

Stepp et al. 2010 [35]	Case-control	18 normal and 18 patients with vocal nodules	Reading, spontaneous speech	Double-differential	(1) Thyrohyoid, omohyoid, and sternohyoid (2) Cricothyroid and sternohyoid (contralateral)	NIBcoh	Significant decrease in NIBcoh in patients compared to healthy speakers

Stepp et al. 2011 [40]	Repeated measures	10 normal	Reading, spontaneous speech	Double-differential	(1) Thyrohyoid, omohyoid, and sternohyoid (2) Cricothyroid and sternohyoid (contralateral)	NIBcoh	Significant reduction of NIBcoh during mimicking hyperfunctional voice

RMS: root mean squared; MTD: muscle tension dysphonia; EMG: electromyograghy; sEMG: surface electromyograghy; SCM: sternocleidomastoid; NIBcoh: neck intermuscular beta coherence.
